# Synthesis, Characteristics, and Effect of Zinc Oxide and Silver Nanoparticles on the In Vitro Regeneration and Biochemical Profile of Chrysanthemum Adventitious Shoots

**DOI:** 10.3390/ma15228192

**Published:** 2022-11-18

**Authors:** Alicja Tymoszuk, Natalia Sławkowska, Urszula Szałaj, Dariusz Kulus, Małgorzata Antkowiak, Jacek Wojnarowicz

**Affiliations:** 1 Laboratory of Ornamental Plants and Vegetable Crops, Faculty of Agriculture and Biotechnology, Bydgoszcz University of Science and Technology, Bernardyńska 6, 85-029 Bydgoszcz, Poland; 2 ExPlant Student Association, Faculty of Agriculture and Biotechnology, Bydgoszcz University of Science and Technology, Bernardyńska 6, 85-029 Bydgoszcz, Poland; 3 Laboratory of Nanostructures, Institute of High Pressure Physics, Polish Academy of Science, Sokolowska 29/37, 01-142 Warsaw, Poland; 4 Department of Organic Agriculture and Environmental Protection, Institute of Plant Protection–National Research Institute, Władysława Węgorka 20, 60-318 Poznań, Poland

**Keywords:** adventitious organogenesis, *Chrysanthemum × morifolium* (Ramat.) Hemsl., in vitro culture, nanotechnology, plant metabolites, regeneration efficacy, zinc oxide, silver, nanoparticles

## Abstract

Studies on nanoparticles’ effects on plants are relevant for horticulture. This study aimed to test the influence of zinc oxide submicron particles (ZnO SMPs), zinc oxide nanoparticles (ZnO NPs), and zinc oxide nanoparticles combined with silver nanoparticles (ZnO+1%Ag NPs) applied at 100 and 500 mg·L^−1^ on the regeneration and biochemical activity of adventitious shoots in *Chrysanthemum × morifolium* (Ramat.) Hemsl. ‘UTP Burgundy Gold’ and ‘UTP Pinky Gold’. The original microwave solvothermal synthesis and characteristics of the ZnO samples were described. Internodes were cultured on the MS medium with 0.6 mg∙L^−1^ 6-benzylaminopurine (BAP) and 2 mg∙L^−1^ indole-3-acetic acid (IAA). In ‘UTP Burgundy Gold’, the highest shoot regeneration efficiency was obtained for 100 mg·L^−1^ ZnO SMPs and 500 mg·L^−1^ ZnO NPs treatments (6.50 and 10.33 shoots per explant, respectively). These shoots had high or moderate chlorophyll and carotenoid contents. In ‘UTP Pinky Gold’, the highest shoot number was produced in the control (12.92), for 500 mg·L^−1^ ZnO SMPs (12.08) and 500 mg·L^−1^ ZnO NPs (10.42). These shoots had increased chlorophyll (*a*+*b*)-to-carotenoid ratios. In ‘UTP Pinky Gold’, the ZnO SMPs and ZnO NPs affected the anthocyanins biosynthesis, whereas ZnO + 1%Ag NPs decreased the phenolics accumulation. These results are important for the improvement of chrysanthemum micropropagation.

## 1. Introduction

Nanoparticles (NPs), i.e., small particles of matter between 1 and 100 nanometers in diameter, are characterized by unique physicochemical properties that are directly related to their small size. These properties differ when compared to those possessed by material counterparts at the micrometric scale, often ensuring new applications of existing materials [1]. Engineered NPs are considered to be potential fertilizers, growth enhancers, and pesticides for agriculture [2]. However, exposure of plants to engineered NPs is still not a fully explored area of research and object of discussion. Results of studies on NPs–plant interactions are not only scientifically relevant but, additionally, can be also implemented in horticultural practice to highly increase the efficiency of plant production and/or support breeding programs of new cultivars [3,4].

Micropropagation is a clonal propagation method of plants performed under aseptic and fully controlled laboratory conditions. Inside the culture vessels, plants are grown on synthetic media that contain nutrient formulations of salts and vitamins, sugar, and growth regulator [5]. In such conditions, differentiated somatic cells may dedifferentiate and subsequently redifferentiate into (adventitious) shoots, roots, or embryos. The formation of adventitious shoots (caulogenesis) and roots (rhizogenesis) is specified by the term adventitious organogenesis, whereas somatic embryogenesis refers to the formation of adventitious (somatic) embryos. Adventitious organogenesis may be induced with very high efficiency in tissue culture, representing one of the basic methods of vegetative plant propagation and a valuable tool useful in plant breeding [6]. In modern horticulture, in vitro cultures have been employed for various purposes, e.g., large-scale production of pathogen-free plants, germplasm conservation, or secondary metabolites production [7]. Plant cell and tissue culture systems also provide proper and unbiased growth conditions for studying the independent effects of an elicitor or inhibitor, i.e., ZnO nanoparticles or other nanoparticles, on micropropagation course and efficiency [8].

*Chrysanthemum × morifolium* (Ramat.) Hemsl. (syn. *Chrysanthemum × grandiflorum*/Ramat./Kitam.) is one of the most economically important and favored floricultural crops worldwide, ranking second in the cut flower trade, following the rose. Chrysanthemum is also cultivated as a pot plant and used in gardening and ornamental landscaping. The market demand for chrysanthemum cultivars with new inflorescence characteristics, improved stress tolerance, or quality attributes is increasing annually, being a great challenge for chrysanthemum breeders [9]. Chrysanthemum micropropagation by adventitious shoots regeneration from non-meristematic plant parts (explants), e.g., leaves, internodes, or petals, is a common tool applied in most important breeding methods, which include induced mutagenesis, chimera components separation, transgenesis, and the exploitation of somaclonal variation [10].

Zinc is one of the most significant micronutrients required in plant growth and development, playing an important role in many cellular metabolic processes, being the only metal found in all six enzyme classes (oxidoreductases, lyases, isomerases, transferases, hydrolases, and ligases). Zinc stimulates the biosynthesis of plant chlorophylls and carotenoids, enhancing photosynthetic activity and repairing photosystem II. This element is also an integral part of transcription factors of the zinc finger family. Cell division and differentiation, along with cellular repair and defense mechanisms, are also affected by zinc ion content in plant cells [2,8,11].

Zinc oxide nanoparticles (ZnO NPs) belong to the most broadly produced NPs worldwide [2,12,13]. They are considered a bio-safe material for plants and are used in plant production to stimulate seed germination and plant growth, as well as in plant protection for disease suppression, due to their antimicrobial activity. Nonetheless, both positive and negative effects on the in vitro and in vivo plant growth were reported. These depended on the physicochemical properties of ZnO NPs and acted in a dose-dependent manner [11]. Zinc oxide NPs are suggested to provide plants with near-exact concentrations of required Zn^2+^. However, determining the deficiency/sufficiency/toxicity thresholds of ZnO NPs in culture media is still under investigation [8]. The addition of ZnO NPs (50–150 mg·L^−1^) to the culture medium stimulated callus proliferation, shoot multiplication, and rooting in date palm (*Phoenix dactylifera* L.) [7,14]. ZnO NPs and zinc oxide submicron particles (ZnO SMPs) in the concentration range from 50 to 1600 mg∙L^−1^ stimulated the in vitro germination of onion (*Allium cepa* L.) seeds, without negative effects on the further growth and development of seedlings [15]. The supplementation of culture medium with ZnO NPs at the concentration of 6 and 8 mg∙L^−1^ increased the micropropagation efficiency, as well as the fresh and dry weight of olive (*Olea europaea* L.) microshoots [16]. On the other hand, silver nanoparticles (AgNPs) are one of the most often used nanomaterials in plant science, especially in agriculture and horticulture. Although the influence of AgNPs on plant germination, growth, and metabolism was extensively studied, there is still a deficiency of research concerning the impact of NPs on adventitious organogenesis in ornamental plants [3,4,10].

This study aimed to test, for the first time, the influence of ZnO SMPs, ZnO NPs, and zinc oxide nanoparticles combined with silver nanoparticles (ZnO+1%Ag NPs), used at the concentrations of 100 and 500 mg·L^−1^, on the in vitro regeneration and biochemical activity of adventitious shoots in chrysanthemum ‘UTP Burgundy Gold’ and ‘UTP Pinky Gold’. The enhanced regeneration potential of internode explants resulting from nanoparticles application would be of great importance for multiplication improvement and breeding purposes in chrysanthemum.

## 2. Materials and Methods

### 2.1. Materials Used in the Synthesis of Nanomaterials Samples

The following were used for the synthesis of ZnO NPs and ZnO+1%Ag NPs: zinc acetate dihydrate (Zn(CH_3_COO)_2_∙2H_2_O, Avantor Performance Materials Poland S.A., Gliwice, Poland); silver acetate anhydrous (Ag(CH_3_COO), Chempur, Piekary Śląskie, Poland); ethylene glycol (C_2_H_4_(OH)_2_, Chempur, Piekary Śląskie, Poland); deionized water (H_2_O) (specific conductance below 0.1 μS·cm^−1^). All the chemical substances were analytically pure and used without further purification. Submicron particles of pharmaceutically pure zinc oxide (ZnO SMPs) were purchased from ZM SILESIA SA, Huta Oława, Oława, Poland [17].

### 2.2. Synthesis of NPs

The original microwave solvothermal synthesis procedure applied for the synthesis of ZnO NPs was described in detail in our previous articles [18,19]. In short, Zn(CH_3_COO)_2_∙2H_2_O (35.00 g) was dissolved in C_2_H_4_(OH)_2_ (525 mL) with the use of the hot-plate magnetic stirrer (13 min, 70 °C, 450 rpm). Subsequently, the solution was decanted into a polypropylene (PP) bottle and sealed. Once the solution reached the room temperature, a water content analysis was carried out (Cp_H2O_ = 1.03 wt%) and 2.8929 g of water was added to reach the final water concentration of 1.5 wt%. The precursor solution was subjected to microwave heating in the microwave reactor MSS2 (270 mL, 12 min, 4 bar, 3 kW, 2.45 GHz), IHPP PAS (Warsaw, Poland), ITeE-PIB (Radom, Poland), ERTEC (Wroclaw, Poland) [20]. After cooling the reactor chamber down, the post-reaction suspended matter was removed, centrifuged and thoroughly rinsed four times with deionized water. After each rinsing, the sample was centrifuged again. Homogeneous water suspension was obtained from the white paste and was frozen rapidly using liquid nitrogen and dried in the freeze dryer. Thus, the obtained sample was named ZnO NPs.

In order to obtain a sample with the nominal composition of 99%·ZnO+1%·Ag NPs, the microwave solvothermal co-synthesis procedure, described in detail in our previous article [21], was applied. In short, (Zn(CH_3_COO)_2_∙2H_2_O (35.0000 g) and Ag(CH_3_COO) (0.2664 g) were poured into C_2_H_4_(OH)_2_ (525 mL) and stirred with the use of the hot-plate magnetic stirrer (70 °C, 450 rpm) until the reagents dissolved. Subsequently, the solution was decanted into a PP bottle and sealed. Once the solution reached the room temperature, a water content analysis was carried out (Cp_H2O_ = 1.06 wt%) and 2.7249 g of water was added to reach the final water concentration of 1.5 wt%. The precursor solution was subjected to microwave heating in the microwave reactor, MSS2 (270 mL, 12 min, 1 bar, 3 kW, 2.45 GHz), IHPP PAS (Warsaw, Poland), ITeE-PIB (Radom, Poland), ERTEC (Wroclaw, Poland) [20]. After cooling the reactor chamber down, the post-reaction suspended matter was removed, centrifuged and thoroughly rinsed four times with deionized water. After each rinsing, the sample was centrifuged again. Homogeneous water suspension was obtained from the greyish paste and was frozen rapidly using liquid nitrogen and dried in the freeze dryer. After drying, the sample was named ZnO+1%Ag NPs.

### 2.3. Characterization of ZnO Sample

The tests of the ZnO NPs, ZnO+1%Ag NPs and ZnO SMPs samples were carried out in the Laboratory of Nanostructures (IHPP PAS, Warsaw, Poland), which holds accreditation no. AB 1503 [22]. A detailed description of the test procedures used can be found in our previous paper [23].

X-ray powder diffraction (XRD) patterns were collected on an X-ray diffractometer X’Pert PRO with CuKα radiation (Panalytical, Almelo, The Netherlands). The morphology of the obtained samples was analyzed using the scanning electron microscope ULTRA PLUS (ZEISS, Oberkochen, Germany). The skeleton density (pycnometric density) of NPs samples was tested using an AccuPyc II 1340 helium pycnometer (FoamPyc V1.06, Micromeritics^®^, Norcross, GA, USA). The specific surface area was measured by the Brunauer-Emmett-Teller (BET) method with the use of a Gemini 2360 (V 2.01, Micromeritics^®^, Norcross, GA, USA). The Zn and Ag content in the ZnO+1%Ag NPs was determined by the energy dispersive spectrometry (EDS) method with the use of a Quantax 400 (Bruker, Billerica, MA, USA). The presented results of Zn and Ag content are average values calculated based on six analyses of different areas of the sample, which was pressed to the form of pastille. The water content (wt%) in the glycol solution samples was measured with the use of a Cou-Lo AquaMAX KF (GR Scientific, Bedford, UK), which applies the Karl Fischer method.

The average crystallite size was calculated using the Scherrer equation [18] and the Nanopowder XRD Processor Demo web application [24,25]. The crystallite size distribution was obtained using the Nanopowder XRD Processor Demo web application. The average particle size was calculated with the use of the skeleton density results and the specific surface area results [18].

### 2.4. Chrysanthemum In Vitro Culture–Establishment, Nanoparticles Treatment, Ambient Conditions

The composition of the medium for the in vitro culture was optimized in our previous studies on adventitious organogenesis in various chrysanthemum cultivars [3,4,10]. A modified Murashige and Skoog (MS) medium [26], with the content of calcium and iron increased by half, was used for micropropagation. The medium was additionally supplemented with 30 g·L^−1^ sucrose and solidified with 8 g·L^−1^ Plant Propagation LAB-AGAR^TM^ (BIOCORP, Warsaw, Poland). Plant growth regulators (PGRs): 0.6 mg∙L^−1^ 6-benzylaminopurine (BAP) and 2 mg∙L^−1^ indole-3-acetic acid (IAA) (Sigma-Aldrich, St. Louis, MO, USA) were added to the medium to stimulate the regeneration of adventitious shoots. The pH level of the medium was adjusted to 5.8 with the use of HCl and NaOH (Chemia Sp. z o.o. Bydgoszcz, Poland) prior to medium sterilization. Approximately 40 mL of the medium was poured into 350-mL glass jars, which were next sealed with a plastic lid, and autoclaved at 105 kPa and 121 °C for 20 min.

Two *Chrysanthemum × morifolium* (Ramat.) Hemsl. cultivars: ‘UTP Burgundy Gold’ and ‘UTP Pinky Gold’, were used in the experiment. Aseptic internodes, dissected from plantlets cloned previously with the single-node method on the modified MS medium without plant growth regulators, were used as explants. Four explants per each culture jar were placed horizontally on the medium. Immediately after the inoculation onto the medium, the explants were treated with zinc oxide submicron particles suspension (ZnO SMPs), zinc oxide nanoparticles suspension (ZnO NPs), or zinc oxide and silver nanoparticles suspension (ZnO+1%Ag NPs), at the concentration of 100 or 500 mg·L^−1^ selected on the basis of preliminary research (data not shown). The tested suspensions were sterilized in an autoclave, and before application, placed for 30 min in the Elmasonic S80(H) Ultrasonic Cleaner with the ultrasonic frequency of 37 kHz and the effective ultrasonic power of 150 W (Elma Schmidbauer GmbH, Singen, Germany) to achieve proper dispersion of particles. Suspensions were poured onto the culture medium with an automatic pipette with a sterile tip, 2 mL of each suspension, at each tested concentration per culture jar. Non-treated explants were used as the control object.

In vitro cultures were kept in the growth room at ambient conditions: temperature 23 ± 1 °C, 16/8 h light/dark photoperiod provided by Philips TLD 36W/54 fluorescent lamps (Koninklijke Philips Electronics N.V., Eindhoven, the Netherlands) emitting cool daylight with the photosynthetic photon flux density set at 35 μmol·m^−2^·s^−1^.

Observations of the dynamics of adventitious shoots regeneration were performed weekly, for eight successive weeks. Next, the share of explants forming shoots, and the mean number of shoots per one inoculated explant, were estimated in the ninth week of culture to assess the regeneration efficacy. 

### 2.5. Biochemical Analysis of Regenerated Shoots

The regenerated adventitious shoots were subjected to biochemical analysis to estimate the content of plant metabolites (chlorophyll *a*, chlorophyll *b*, carotenoids, anthocyanins, phenolic compounds). The whole shoots (1–3 cm in length) were used to prepare fresh weight (FW) samples. As for the cultivar ‘UTP Burgundy Gold’, the extraction of anthocyanins and phenolic compounds was not performed due to the limited amount of plant material obtained from some experimental treatments.

Chlorophylls and carotenoids were extracted using 100 mg FW samples and 100% acetone (Chemia Sp. z o.o., Bydgoszcz, Poland) according to Lichtenthaler’s [27] procedure. Anthocyanins were extracted using 200 mg FW samples and methanol containing 1% HCl (*v*/*v*) (Chemia Sp. z o.o., Bydgoszcz, Poland), as described by Harborne [28]. The same extract was used for the analysis of the total phenolic content according to use of the Folin–Ciocalteau protocol [29], based on gallic acid (Sigma-Aldrich, St. Louis, MO, USA), as the calibration standard.

The spectrophotometric analyses were performed in the NanoPhotometer^®^ NP80 (Implen, München, Germany) at specific wavelengths (λ_max_): 530 nm, 645 nm, 662 nm, 470 nm, and 765 nm for anthocyanins (cyanidin-3-glucoside), chlorophyll *a*, chlorophyll *b*, carotenoids, and phenolic compounds, respectively. The content of the plant metabolites was calculated in milligrams per one gram of sample fresh weight (mg·g^−1^ FW).

### 2.6. Statistical Analysis

The experiment was set up in a completely randomized design. Each experimental object consisted of 3 jars (12 explants in total). One explant was considered as one repetition. Biochemical analyses were repeated six times. The obtained data were presented as mean ± standard deviation (SD) and subjected to one-way analysis of variance (ANOVA) and a post hoc Fisher test at the significance level of *p* ≤ 0.05. For data expressed as a percentage, the Freeman–Tukey transformation was used. All statistical analyses were performed with the use of the Statistica 13.3 software (StatSoft Polska, Cracow, Poland).

## 3. Results and Discussion

### 3.1. Morphology of ZnO Samples

**Figure 1:**
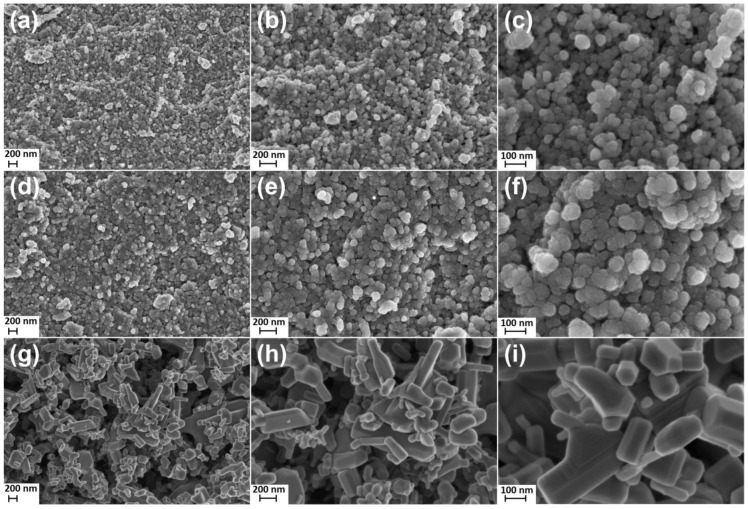
SEM images of samples: (**a**–**c**) ZnO NPs; (**d**–**f**) ZnO+1%Ag NPs; (**g**–**i**) ZnO SMPs. (**a**–**i**) images taken with the immersion lens detector. Image magnifications: (**a**,**d**,**g**) 50,000×; (**b**,**e**,**h**) 100,000×; (**c**,**f**,**i**) 250,000×.

Figure 1 contains the results of the SEM analysis, which present the morphology of the ZnO NPs (Figure 1a–c) sample, the ZnO+1%Ag NPs sample (Figure 1d–f) and the ZnO SMPs (Figure 1g–i) sample used in the tests. The SEM images were created with three different magnifications: 50,000× (Figure 1a,d,g), 100,000× (Figure 1b,e,h) and 250,000× (Figure 1c,f,i) in order to determine the shape, homogeneity and size of particles.

The ZnO NPs sample (Figure 1a–c) and the ZnO+1%Ag NPs sample (Figure 1d–f) were characterized by homogeneity of particle shape and size. ZnO NPs and ZnO+1%Ag NPs obtained by the microwave method had a spherical shape and their size ranged from 15 nm to 70 nm. The obtained NPs samples confirm that products of microwave solvothermal synthesis of nanomaterials are characterized by homogeneity of shape, homogeneity of size and a narrow particle size distribution. It must be borne in mind that it is the synthesis method applied that determines the morphology of the obtained nano-ZnO product [30]. Our original synthesis procedure makes it possible to obtain an identical morphology of particles, not only for ZnO NPs [18,19] and ZnO+Ag NPs [21], but also for Co-doped ZnO NPs [23,31], Mn-doped ZnO NPs [32] as well as Co-Mn codoped ZnO NPs [33]. The method of ZnO+Ag NPs synthesis developed by us is distinguished by three essential features compared to other methods reported in the literature [34,35,36,37,38,39,40,41,42,43,44]. Firstly, we eliminated the stage of sample heating/calcination, which results in particle growth and contributes to the formation of agglomerates/aggregates. Secondly, NPs samples are subjected to the freeze-drying process, which considerably reduces particle agglomeration. Thirdly, the end product is a dry, loose and fluffy NPs powder, from which it is easy to obtain a water suspension. A commercial sample of ZnO SMPs, visible in the SEM images (Figure 1g–i), was produced by the indirect French process where metallic zinc was the input raw material [45,46]. The ZnO SMPs sample was composed of rod-shaped particles, hexagonal structures and structures characterized by a heterogeneous shape. Particles with the highest size, ranging from approx. 100 nm to approx. 2000 nm, prevailed in the ZnO SMPs sample. It can be stated, based on the SEM image (Figure 1i) that the ZnO SMPs sample was composed of aggregates of particles, i.e., permanently joined particles on their contact surface. The morphology of the sample of commercial ZnO SMPs was typical of ZnO produced by the indirect French process, as confirmed by the work of Moezzi et al. [46].

**Figure 2:**
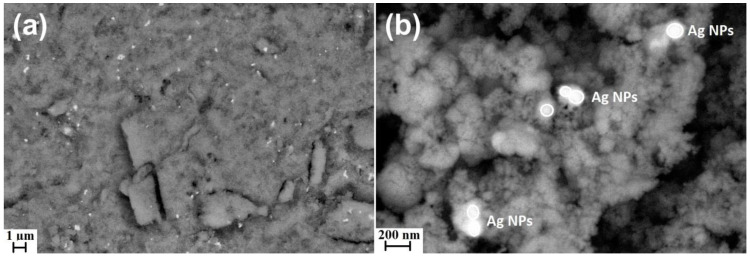
SEM images of ZnO+1%Ag NPs. (**a**,**b**) images taken with the angle-selective backscatter detector. Image magnifications: (**a**) 10,000×; (**b**) 100,000×.

Figure 2 presents the results of SEM analyses with the use of the angle-selective backscatter (AsB) detector for the ZnO+1%Ag NPs sample. The AsB detector made it possible to achieve a clear composition contrast between zinc oxide and silver, which enables an easy identification of Ag nanoparticles in the SEM images. The SEM image (Figure 2a) confirms a homogeneous distribution of Ag particles in the ZnO+1%Ag NPs sample. It must be underlined that obtaining a homogeneous mixture of ZnO NPs with Ag NPs in the nanoscale is technologically very difficult. The microwave solvothermal co-synthesis method makes it possible to obtain a homogeneous mixture of two products of synthesis, ZnO and Ag, during a single process. It must be emphasized that in the microwave solvothermal co-synthesis reaction, a solution of reagents was used, in which an identical concentration of ZnO NPs and Ag NPs precipitated in the whole volume during the temperature increase. The SEM results (Figure 2) prove that spherical Ag NPs approx. 70–100 nm in size occur in the form of agglomerates/aggregates composed of at least two/three nanoparticles. ZnO NPs form a physical barrier that restricts further Ag NPs agglomeration/aggregation processes in a dry sample.

### 3.2. Phase Composition of ZnO Samples

**Figure 3:**
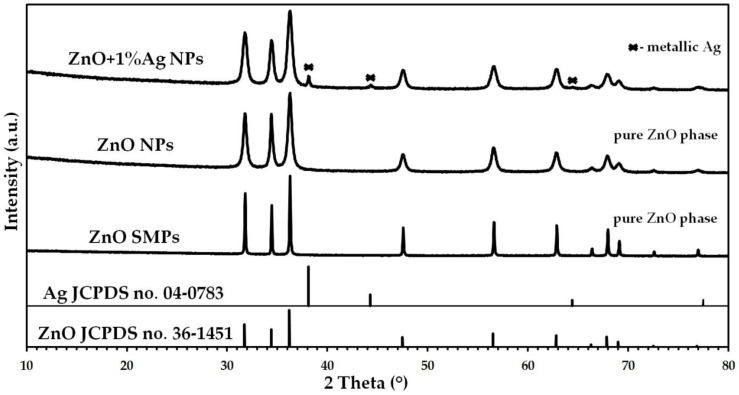
X-ray diffraction patterns of ZnO NPs, ZnO+1%Ag NPs and ZnO SMPs.

The crystalline structure of the ZnO samples was tested by the XRD method, while the results of the analyses are presented in Figure 3. The XRD result for the reference sample of hexagonal wurtzite structure ZnO (JCPDS card no. 36-1451) is characterized by the presence of diffraction peaks with the following 2 theta values: 31.77°, 34.42°, 36.25°, 47.54°, 56.60°, 62.86°, 66.38°, 67.96°, 69.10°, 72.56° and 76.95° [47]. The XRD result for the reference sample of the cubic crystal structure Ag (JCPDS card no. 04-0783) is characterized by the presence of diffraction peaks with the following 2 theta values: 38.12°, 44.28°, 64.43° and 77.47° [48]. All diffraction peaks obtained in the XRD results for the ZnO NPs and ZnO SMPs samples originated from the hexagonal phase ZnO with the wurtzite structure (Figure 3). No presence of foreign crystalline phases was found in the ZnO NPs and ZnO SMPs samples within the detection limits of the applied XRD method. The XRD results indicated the presence of diffraction peaks originating from hexagonal phase ZnO with the wurtzite structure and cubic crystal structure of Ag in the ZnO+1%Ag NPs sample (Figure 3). The ZnO+1%Ag NPs sample, similarly to the others, did not contain any crystalline impurities. Analogous XRD results for ZnO+Ag NPs samples are reported by other research groups [34,35,36,44,49,50].

### 3.3. Density, Specific Surface Area, and Average Size and Crystallite Size Distribution

**Figure 4:**
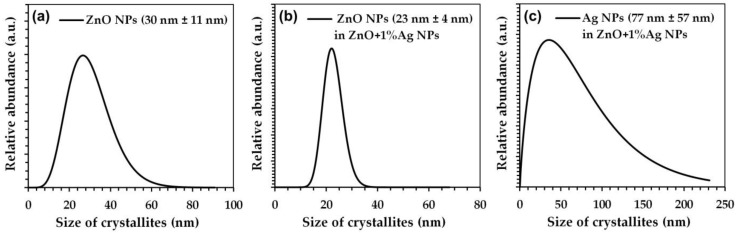
Crystallite size distributions of: (**a**) ZnO NPs, (**b**) ZnO NPs in ZnO+1%Ag NPs, (**c**) Ag NPs in ZnO+1%Ag NPs.

The summary of results for the sample characterization is included in Table 1. The skeleton density values for the ZnO NPs sample and the ZnO+1%Ag NPs sample were 5.09 ± 0.06 g·cm^−3^ and 5.05 ± 0.05 g·cm^−3^, respectively. Taking into account the value of the standard deviation for the obtained skeleton density results, it can be stated that no significant differences were observed in these results. The specific surface area values for ZnO NPs and ZnO+1%Ag NPs were 48.4 m^2^·g^−1^ and 44.4 m^2^·g^−1^, respectively. The smaller specific surface area of the ZnO+1%Ag NPs sample resulted from the presence of Ag NPs in its composition. Wu et al. [50] obtained an identical difference in the specific surface area value, 4 m^2^·g^−1^, between the samples obtained by the solvothermal method (ZnO nanorods −80.3 m^2^·g^−1^, ZnO+(5 mol%)Ag nanocomposite −75.9 m^2^·g^−1^). This might indicate that during the solvothermal co-synthesis of ZnO+Ag there is a close correlation between the nanoproducts, which should be explained by researching and verifying the co-synthesis mechanism. The average particle sizes, calculated on the basis of the specific surface area results for the ZnO NPs sample and the ZnO+1%Ag NPs sample, were 25 ± 2 nm and 27 ± 2 nm, respectively. The average ZnO crystallite sizes, calculated on the basis of the Scherrer equation for the ZnO NPs sample and the ZnO+1%Ag NPs sample, were 31 ± 8 nm and 22 ± 3 nm, respectively. The results based on the Scherrer equation coincided for these samples with the results obtained from the Nanopowder XRD Processor Demo application (Table 1). The average size of Ag crystallites contained in the ZnO+1%Ag sample was, depending on the method applied, 45 ± 20 nm (Scherrer equation) and 77 ± 57 nm (Nanopowder XRD Processor Demo). The obtained results prove that, in the microwave solvothermal co-synthesis of the ZnO+1%Ag NPs sample, the reaction of the Ag NPs synthesis exerted a significant impact on the change in the average size of ZnO NPs crystallites and in their size distribution (Figure 4). The ZnO crystallite size distribution in the ZnO NPs size ranged from approx. 7 nm to approx. 70 nm, while for the ZnO+1%Ag NPs sample it ranged from approx. 11 nm to approx. 40 nm (Figure 4). The Ag crystallite size distribution in the ZnO+1%Ag NPs sample, in turn, ranged from several nm to 230 nm and was caused by the impact of the simultaneous reaction of ZnO NPs synthesis on the formation of Ag NPs. The commercial ZnO SMPs sample had the skeleton density of 5.59 ± 0.03 g·cm^−3^, the specific surface area of 4.5 m^2^·g^−1^ and the average particle size of 240 ± 30 nm. The difference between the density of the ZnO SMPs sample and of the ZnO NPs sample results mainly from the particle size difference. The impact of the particle size on the particle properties, such as density and specific surface area, is common knowledge and does not concern only zinc oxide [51], but also hydroxyapatite [52] and zirconium dioxide [53]. The main cause of the difference between the values of skeleton density of the ZnO SMPs and ZnO NPs samples is the presence of an amorphous phase in the ZnO NPs sample [51]. Unfortunately, the skeleton density test is still not very popular among research groups that deal with the obtaining and characterising of ZnO+Ag NPs, which is proved by the lack of skeleton density results reported in the published papers [34,35,36,37,38,39,40,41,42,43,44,49,50]. Based on the comparison of the SEM results (Figure 1 and Figure 2) and on the average crystallite size results, it can be stated that the ZnO SMPs sample was composed of polycrystalline particles. The same conclusion concerning polycrystallinity can be drawn also for the Ag particles that were present in the ZnO+1%Ag NPs sample.

### 3.4. Chemical Composition of ZnO Samples

The purity of the ZnO SMPs sample meets the requirements of the European Pharmacopoeia, while the thresholds of trace impurities for the pharmaceutical zinc oxide used are provided in the producer’s catalogue [17]. The purity of the ZnO NPs sample was equal to the analytical purity class, which resulted from the quality of the reagents used and from the performance of the synthesis in the reaction chamber made of polytetrafluoroethylene (PTFE) [20,30]. Table 2 summarises the results of the quantitative chemical analysis performed by the EDS method for the ZnO+1%Ag NPs sample. The nominal composition of the ZnO+1%Ag NPs sample, arising from the quantity of reagents used for the co-synthesis reaction, was as follows: 99%·ZnO+1%·Ag. The actual composition of the ZnO+1%Ag NPs sample, determined thanks to the EDS analysis, was as follows: 99.05%·ZnO+0.95%·Ag. After converting the actual quantitative contents of zinc and silver to weight contents, it was determined that a 100 mg ZnO+1%Ag NPs sample contained 98.75 mg of ZnO NPs and 1.25 mg of Ag NPs.

### 3.5. Adventitious Shoots Regeneration and Biochemical Assay of Phytochemicals

**Figure 5:**
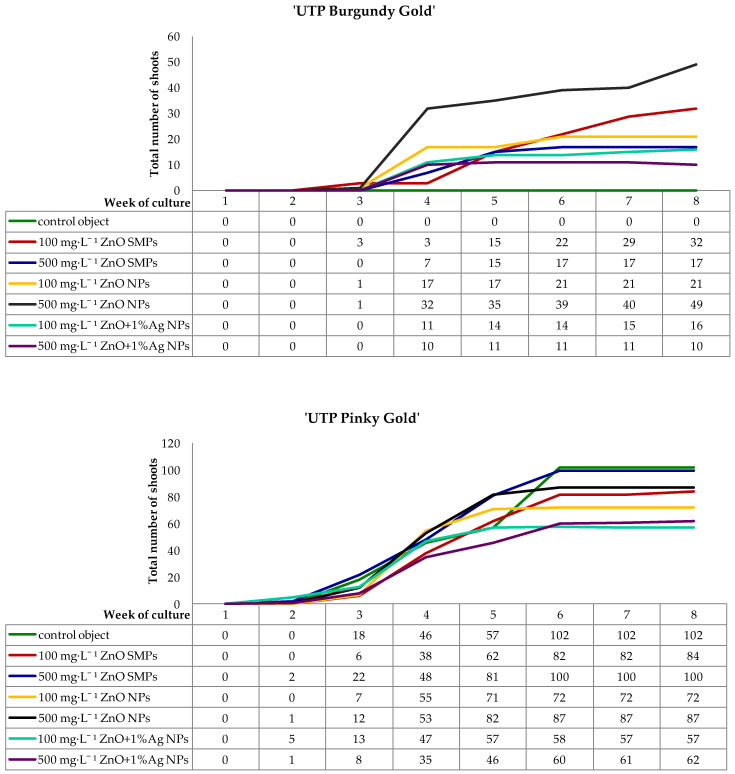
Dynamics of adventitious shoots regeneration on *Chrysanthemum × morifolium* ‘UTP Burgundy Gold’ and ‘UTP Pinky Gold’ internodes cultured on the modified MS medium with 0.6 mg·L^−1^ BAP and 2 mg·L^−1^ IAA, depending on the experimental treatment.

**Figure 6:**
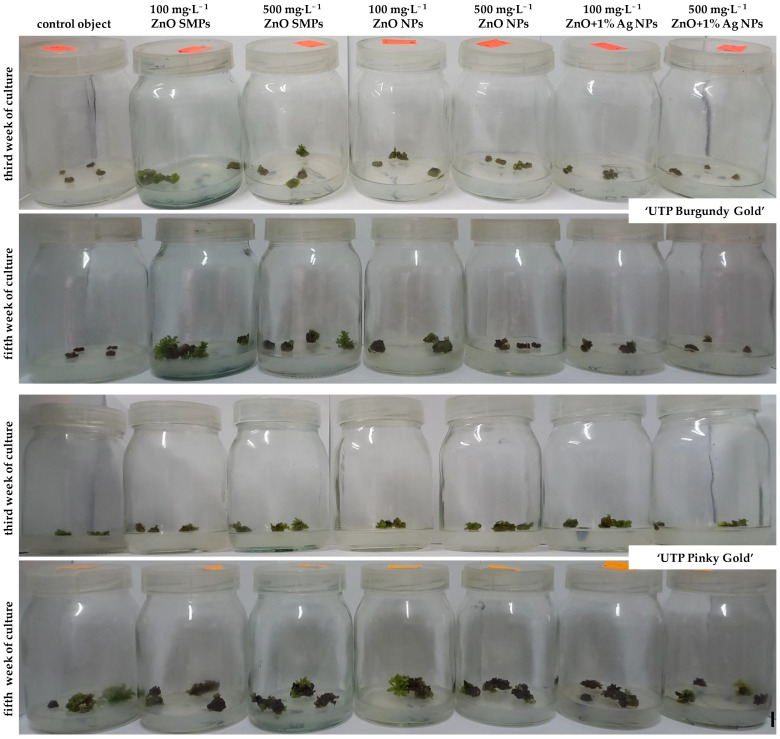
Regeneration of adventitious shoots on *Chrysanthemum × morifolium* ‘UTP Burgundy Gold’ and ‘UTP Pinky Gold’ internodes cultured on the modified MS medium with 0.6 mg·L^−1^ BAP and 2 mg·L^−1^ IAA, depending on the experimental treatment and culture week; bar = 1 cm.

During the first week of culture, all inoculated internodes enlarged visually their volume as a result of absorbing water and medium components. The regeneration of green callus, first in the area of cutting edge and next on the whole surface of inoculated internodes, began in the second week of culture. Chrysanthemum ‘UTP Pinky Gold’ proliferated callus more intensively than the ‘UTP Burgundy Gold’ cultivar. Therefore, the formation of adventitious shoots occurred indirectly via calli (Figure 5 and Figure 6).

In ‘UTP Burgundy Gold’ cultivar, the regeneration of first shoots was observed in the third week of culture on internodes treated with 100 mg·L^−1^ ZnO SMPs and with ZnO NPs applied at both tested concentrations. During the next week, shoots appeared on internodes in other experimental treatments, except for the control object, where no regeneration was observed during the whole culture period. Interestingly, the highest increase in the number of shoots was reported in the fourth week, especially on internodes treated with ZnO NPs and ZnO+1%Ag NPs, irrespective of the tested concentration. As for the ZnO SMPs treatments, the regeneration was most intensive in the fifth week. During the following culture weeks, the dynamic of shoot regeneration was moderate. Finally, in the eighth week, the highest number of 49 shoots was reported on the internodes treated with 100 mg·L^−1^ ZnO SMPs, while the lowest number of 10 shoots was observed on the 500 mg·L^−1^ ZnO+1%Ag NPs-treated explants (Figure 5 and Figure 6).

As for chrysanthemum ‘UTP Pinky Gold’, regeneration of the first adventitious shoots was observed one week earlier than in ‘UTP Burgundy Gold’, i.e., in the second culture week, in the following treatments: 500 mg·L^−1^ ZnO SMPs, 500 mg·L^−1^ ZnO NPs, 100 mg·L^−1^ and 500 mg·L^−1^ ZnO+1%Ag NPs. In the third week, the formation of shoots was also reported in other experimental combinations. A high increase in the number of appearing shoots was found in the fourth week of culture, irrespective of the experimental treatment. An intensive regeneration of shoots was also reported between the fourth and sixth culture weeks in the control object and for the treatments with ZnO SMPs, and between the fourth and fifth weeks for ZnO NPs and ZnO+1%Ag NPs treatments. Finally, on the control explants and the explants treated with 500 mg·L^−1^ ZnO SMPs, the numbers of observed shoots in the eighth week were 102 and 100, respectively, while the numbers of shoots produced on explants treated with 100 mg·L^−1^ and 500 mg·L^−1^ ZnO+1%Ag NPs were only 57 and 62, respectively (Figure 5 and Figure 6).

Significant differences were found between the two tested chrysanthemum cultivars in terms of their capability for adventitious organogenesis and response to specific experimental treatments (Table 3).

In chrysanthemum ‘UTP Burgundy Gold’, the application of all tested material samples (ZnO SMPs, ZnO NPs, ZnO+Ag NPs) positively affected the ability of internode explants to form shoots and the efficiency of shoot regeneration as compared to the control object, where no shoot regeneration occurred. Nevertheless, the share of explants forming shoots ranged from 41.67% and 50% for 100 mg·L^−1^ and 500 mg·L^−1^ ZnO+1%Ag NPs treatments, respectively, up to 91.67% for 100 mg·L^−1^ and 500 mg·L^−1^ ZnO NPs treatments. Internodes cultured on the medium with 500 mg·L^−1^ ZnO NPs produced the highest number of shoots (10.33). However, the use of the same material sample at a lower concentration of 100 mg·L^−1^ yielded a significantly lower number of shoots (2.42). A high efficiency of shoot formation was also obtained when explants were treated with 100 mg·L^−1^ ZnO SMPs (6.50 shoots per explant). On the contrary, a low efficiency of shoot regeneration was found for the ZnO+1%Ag NPs sample (0.83–1.33) and for 500 mg·L^−1^ ZnO SMPs (1.42) (Table 3).

The share of ‘UTP Pinky Gold’ explants regenerating shoots was very high, ranging from 91.67% in the control and in 100 mg·L^−1^ ZnO+1% Ag NPs treatments, up to 100% in other experimental combinations. The highest efficiency of shoot regeneration was found for the control internodes (12.92 shoots per explant), with an application of 500 mg·L^−1^ ZnO SMPs (12.08) and 500 mg·L^−1^ ZnO NPs (10.42), whereas internodes in other experimental treatments produced 5.83–7.83 adventitious shoots (Table 3).

Adventitious organogenesis is a powerful multipurpose tool in plant biotechnology. The development of a competent system of in vitro organogenesis is the first step for numerous scientific programs [54]. Researchers are constantly searching for new, cheap, and easily accessible medium additives to improve the efficacy of tissue culture systems [55]. Due to their broad spectrum of action, nanomaterials are an interesting object of study [56].

One of the main limitations of traditional and modern breeding programs, multiplication methods, and conservation of genetic resources is the low regeneration frequency of plants. Increasing the regeneration efficiency of chrysanthemum ‘UTP Burgundy Gold’ from nil to almost 100% is a major breakthrough that opens new possibilities in experimental biology studies. Our research confirmed the positive impact of zinc nanoparticles and submicron particles on the in vitro plant development, although with different intensity and efficiency, as was reported previously in analysis of date palm [14] and onion [15]. Zinc is one of the essential micronutrients that stimulates auxin production and plant growth overall [8], which partially explains the results obtained here. After all, the NPs’ impact on plants strongly depends on the size, shape, surface area, surface coatings, concentration, type of synthesis, and several other factors [10]. The method of application of material samples used in the present study is also of significant importance and novelty. In the previous papers, nanomaterials were literally added into the medium [1,4,57]. Conversely, in our study the colloids were poured onto the surface of the medium, making them more available for the explants (zinc and silver particles could only penetrate into the outer layer of the medium). When adding nanomaterials into the medium, particles sediment into its deepest layers and become less accessible for the plants. Generally, the effects of ZnO SMPs and ZnO NPs on shoot regeneration in ‘UTP Pinky Gold’ chrysanthemum were comparable. The observed variability in regeneration efficiency seemed to be more related to the used concentration of zinc samples than to particles size. Similarly, in the in vitro study of onion, different sizes of zinc particles only affected the initial zinc solubility but did not contribute to the change in the final concentration of zinc ions in the medium [15]. On the other hand, in ‘UTP Burgundy Gold’, the best regeneration results were found for 100 mg·L^−1^ ZnO SMPs and 500 mg·L^−1^ ZnO NPs. The heterogenous response of this cultivar to the tested treatments may be genotype-specific and requires further study.

Interestingly, in both tested cultivars, the use of samples containing 1% AgNPs did not increase the explants’ regeneration efficiency considerably as compared to the other treatments. Silver nanoparticles, applied as a homogenous colloid at the concentration of 50 and 100 mg·L^−1^, also inhibited shoot regeneration on internodes and leaf explants in other studies on chrysanthemum, and their use resulted in the induction of phenotypic and genetic variability for breeding purposes [3,4,10]. Likewise, AgNPs’ application at the concentration of 10 and 30 mg·L^−1^ hampered the regeneration of adventitious roots in chrysanthemum and gerbera (*Gerbera × jamesonii* H. Bol.) [10]. Perhaps, the addition of gold nanoparticles would be more effective, as reported in bleeding heart (*Lamprocapnos spectabilis* (L.) Fukuhara) [58], gerbera and Cape Primrose (*Streptocarpus* × *hybridus* Voss.) [10]. Alternatively, different types of nanoparticles, i.e., cuprum or platinum, could be more effective. Nevertheless, the possible use of ZnO NPs+1%Ag NPs in chrysanthemum breeding should be further exploited.

**Figure 7:**
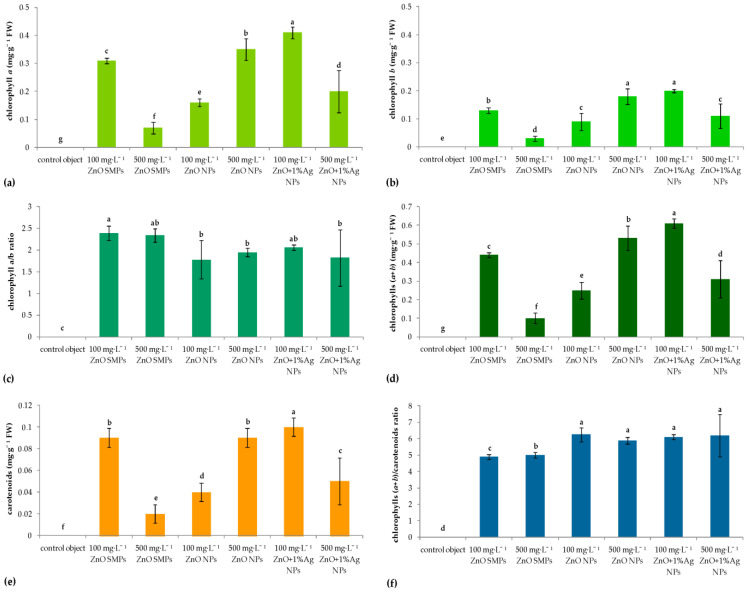
Content of metabolites (chlorophyll *a*, chlorophyll *b*); (**a**–**d**), carotenoids; (**e**,**f**) in adventitious shoots of *Chrysanthemum × morifloium* ‘UTP Burgundy Gold’ regenerated from internodes cultured in vitro for 9 weeks on the modified MS medium with 0.6 mg·L^−1^ BAP and 2 mg·L^−1^ IAA, depending on the experimental treatment. Means ± SD on graphs followed by the same letter do not differ significantly at *p* ≤ 0.05 (Fisher’s test).

As for the results of biochemical analysis, the highest content of chlorophyll *a*, chlorophyll *b*, total chlorophylls, and carotenoids was found in ‘UTP Burgundy Gold’ shoots regenerated on 100 mg·L^−1^ ZnO+1% Ag NPs-treated internodes. Nevertheless, the application of the same sample, but at 500 mg·L^−1^, resulted in a significant decrease in the content of these metabolites compared to the lower concentration. Shoots from the treatments, which provided the best results in terms of regeneration efficiency, i.e., 100 mg·L^−1^ ZnO SMPs and 500 mg·L^−1^ ZnO NPs, were characterized by a high or moderate capability for metabolites biosynthesis. Shoots formed on explants treated with 500 mg·L^−1^ ZnO SMPs or 100 mg·L^−1^ ZnO NPs had low contents of chlorophyll *a*, chlorophyll *b*, total chlorophylls, and carotenoids. Interestingly, compared to submicron particle treatments, nanoparticle treatments resulted in a significant increase in the values of the chlorophyll (*a*+*b*)-to-carotenoid ratio and decreasing chlorophyll *a*/*b* ratio (Figure 7).

**Figure 8:**
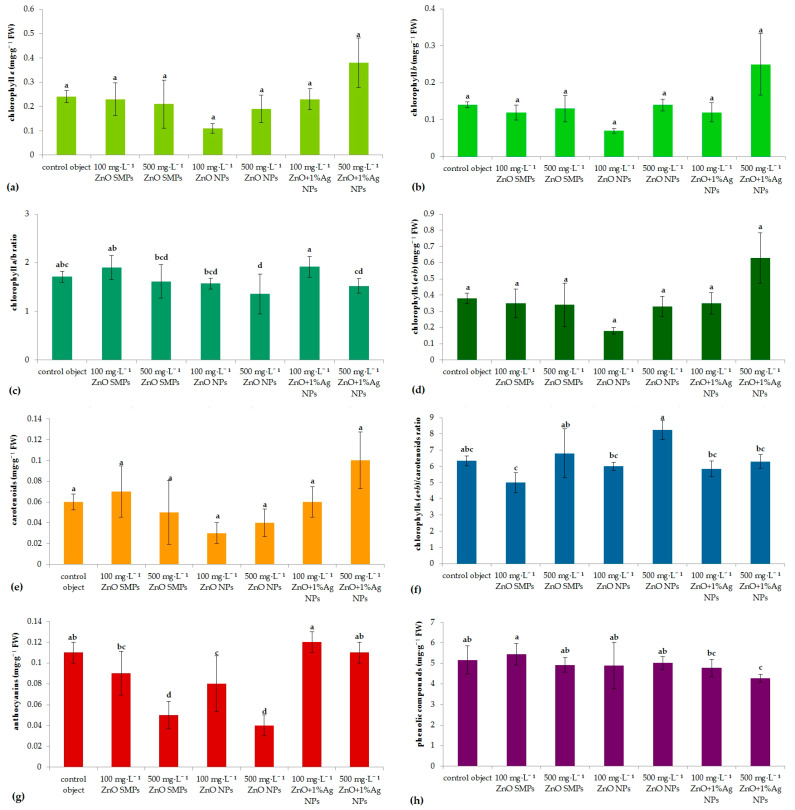
Content of metabolites (chlorophyll *a*, chlorophyll *b*; (**a**–**d**), carotenoids, anthocyanins, phenolic compounds; (**e**–**h**)) in adventitious shoots of *Chrysanthemum × morifloium* ‘UTP Pinky Gold’ regenerated from internodes cultured in vitro for 9 weeks on the modified MS medium with 0.6 mg·L^−1^ BAP and 2 mg·L^−1^ IAA, depending on the experimental treatment. Means ± SD on graphs followed by the same letter do not differ significantly at *p* ≤ 0.05 (Fisher’s test).

On the other hand, in chrysanthemum ‘UTP Pinky Gold’, no significant differences were found between the tested experimental treatments in terms of chlorophyll *a*, chlorophyll *b*, total chlorophylls, and carotenoids contents in the regenerated shoots. The highest (1.71–1.92) chlorophyll *a*/*b* ratios were found in the control object and as a result of 100 mg·L^−1^ ZnO SMPs, and 100 mg·L^−1^ ZnO+1%Ag NPs treatments. Simultaneously, the highest chlorophyll (*a*+*b*)/carotenoid ratios were identified for the control object, 500 mg·L^−1^ ZnO SMPs, and 500 mg·L^−1^ ZnO NPs samples. Treatment with ZnO SMPs and ZnO NPs, especially at a higher concentration, negatively affected the ability of shoots to synthesize anthocyanins compared to the control object and application of ZnO+1%Ag NPs. Interestingly, the content of phenolic compounds in the shoots was similar in the control and ZnO SMPs/ZnO NPs treatments, whereas the use of material samples with silver nanoparticles suppressed the biosynthesis of these compounds (Figure 8).

Nanoparticles can significantly affect the biochemical activity of cells. They can be used as novel elicitors and harvesters to improve bioactive compounds in plants [59,60]. Plant treatment with ZnO NPs can regulate the content of amino acids, organic acids, sugars, glycosides, and metabolites involved in photosynthesis, the antioxidative defense system, and stress resistance [61]. Our studies confirmed the cultivar-specific response of chrysanthemum to zinc nanoparticles and zinc submicron particles, both in terms of regeneration efficiency and biochemical activity.

In a study performed by Al-Mayahi [14], date palm shoots regenerated on media supplemented with 50 and 75 mg·L^−1^ ZnO NPs synthesized more chlorophyll *a* and chlorophyll *b* compared to the control and treatment with 150 mg·L^−1^ ZnO NPs. These results showed that zinc plays a crucial role in increasing the biosynthesis of chlorophylls, and that optimal ZnO NPs treatment can enhance the accumulation of these pigments in plants [14]. Similarly, in chrysanthemum ‘UTP Burgundy Gold’, high or moderate chlorophyll content was found in shoots from the most efficient ZnO NPs/ZnO SMPs treatments in terms of regeneration. ZnO NPs applied at 6 and 18 mg·L^−1^ stimulated the micropropagation of olive (*Olea europea* L.) shoots, as well as enhanced the chlorophyll *a* and *b*, carotenoids, anthocyanins, and total phenolic compound content [16]. On the other hand, ZnO NPs used at the concentrations of 10 and 100 mg·L^−1^, significantly decreased the chlorophyll content in *Vigna radiata* (L.) Wilczek seedlings. As suggested by the authors, the decrease in chlorophyll synthesis might be due to the replacement of the central metal atom of chlorophyll (Mg^2+^) by Zn^2+^, leading to reduced photosynthetic efficiency [62]. Wang et al. [63] found that 200 and 300 mg·L^−1^ ZnO NPs treatments reduced *Arabidopsis* growth by ∼20 and 80%, respectively, in comparison to the control. Pigments measurement showed that chlorophyll *a* and *b* contents were reduced more than 50%, whereas carotenoid content remained largely unaffected in 300 mg·L^−1^ ZnO NPs-treated *Arabidopsis* plants. Consistently, the net rate of photosynthesis, leaf stomatal conductance, intercellular CO_2_ concentration, and transpiration rate were all reduced more than 50% in 300 mg·L^−1^ ZnO NPs-treated plants.

Apparently, chrysanthemum ‘UTP Pinky Gold’ is more tolerant to metal-induced biochemical response of cells, as the contents of chlorophylls and carotenoids did not vary in the plants of this cultivar, irrespectively of zinc form and concentration in the medium. On the other hand, the biosynthesis of anthocyanins, and to a lesser extent also of other polyphenols, was strongly affected by zinc and silver, as the profile of these metabolites has changed in ‘UTP Pinky Gold’ shoots in a wide range, depending on the experimental treatment. Our results are in line with some other studies presenting a varied influence of ZnO NPs on the non-enzymatic antioxidants profile in different plant species [3,4,60]. For example, the polyphenols content was higher in *Perilla frutescens* var. *crispa* f. *purpurea* plants treated with 50 mg·L^−1^ than with 200 mg·L^−1^ ZnO NPs, whereas the treatment with ZnO NPs at 50 and 100 mg·L^−1^ enhanced anthocyanins content as compared with the control and 200 mg·L^−1^ [64]. A study performed on *Capsicum annum* L. showed that accumulation of phenolic compounds in response to 100, 200, and 500 mg·L^−1^ ZnO NPs application differed depending on the tested plant part. The polyphenols content in the plumule was not affected by ZnO NPs, but the increase from 100 to 500 mg·L^−1^ ZnO NPs stimulated the accumulation of these metabolites in the radicles [65].

## 4. Conclusions

This is a maiden study on micropropagation of two new valuable chrysanthemum cultivars: ‘UTP Burgundy Gold’ and ‘UTP Pinky Gold’. The present study confirmed the diversified regenerative potential of non-meristematic explants of even closely related chrysanthemum cultivars. By adding zinc nanoparticles or submicron particles on the surface of the culture medium, it is possible to overcome the recalcitrant potential of cultivars to adventitious organogenesis. For example, in chrysanthemum ‘UTP Burgundy Gold’, the best results in terms of regeneration efficiency were obtained after the treatments with 100 mg·L^−1^ ZnO SMPs and 500 mg·L^−1^ ZnO NPs. Simultaneously, shoots from these combinations had high or moderate contents of chlorophylls and carotenoids as compared to other treatments. On the other hand, in chrysanthemum ‘UTP Pinky Gold’, in shoots from the most efficient experimental treatments, i.e., the control, 500 mg·L^−1^ ZnO SMPs, and 500 mg·L^−1^ ZnO NPs, an increased chlorophylls (*a*+*b*)/carotenoids ratio was reported. Interestingly, in the latter cultivar, the application of ZnO SMPs and ZnO NPs affected negatively the anthocyanins biosynthesis, whereas the treatment with ZnO+1%Ag NPs decreased the accumulation of phenolic compounds. These findings open new research areas in terms of chrysanthemum micropropagation but can also be useful in mutation breeding or genetic transformation. Future studies will focus on a detailed analysis of the genetic stability of regenerants obtained after ZnO NPs and ZnO SMPs treatments. It will be interesting to verify whether the simultaneous application of engineered zinc nanomaterials with physical or chemical mutagens (particularly AgNPs at high concentration) can elevate the effectiveness of mutants regeneration and speed up the breeding process in chrysanthemum and other horticultural crops.

## Figures and Tables

**Table 1:** Characteristics of ZnO samples.

Sample Name	Skeleton Density, ρ_s_ ± σ (g·cm^−3^)	Specific Surface Area, a_s_ (m^2^·g^−1^)	Average Particle Size from SSA BET, d ± σ (nm)	Average Crystallite Size, Scherrer Equation, d ± σ (nm)	Average Crystallite Size, Nanopowder XRD Processor Demo, d ± σ (nm)
ZnO NPs	5.09 ± 0.06	48.4	25 ± 2	31 ± 8	30 ± 11
ZnO+1%Ag NPs	5.05 ± 0.05	44.4	27 ± 2	22 ± 3 (ZnO) 45 ± 20 (Ag)	23 ± 4 (ZnO) 77 ± 57 (Ag)
ZnO SMPs [15]	5.59 ± 0.03	4.5	240 ± 30	124 ± 11	-

D—average size (diameter); SSA—specific surface area; XRD—X-ray powder diffraction.

**Table 2:** Results of the analysis of the chemical composition of ZnO+1%Ag NPs. The method of analysis was energy-dispersive spectrometry (EDS).

Actual Dopant Content (mol%)		Nominal Composition (mol%)	
Zinc	Silver	Zinc	Silver
99.05 ± 0.20	0.95 ± 0.20	99.00	1.00

**Table 3:** Efficiency of adventitious shoots regeneration on *Chrysanthemum × morifolium* ‘UTP Burgundy Gold’ and ‘UTP Pinky Gold’ internodes cultured for 9 weeks on the modified MS medium with 0.6 mg·L^−1^ BAP and 2 mg·L^−1^ IAA, depending on the experimental treatment.

Experimental Treatment	% of Explants Forming Shoots	Number of Shoots Per One Inoculated Explant
	‘UTP Burgundy Gold’	
control object	0.00 c *	0.00 c **
100 mg·L^−1^ ZnO SMPs	75.00 ab	6.50 ± 4.94 ab
500 mg·L^−1^ ZnO SMPs	58.33 ab	1.42 ± 0.90 c
100 mg·L^−1^ ZnO NPs	91.67 a	2.42 ± 1.00 bc
500 mg·L^−1^ ZnO NPs	91.67 a	10.33 ± 5.40 a
100 mg·L^−1^ ZnO+1%Ag NPs	41.67 b	1.33 ± 0.83 c
500 mg·L^−1^ ZnO+1%Ag NPs	50.00 ab	0.83 ± 0.67 c
	**‘UTP Pinky Gold’**	
control object	91.67 a	12.92 ± 7.50 a
100 mg·L^−1^ ZnO SMPs	100.00 a	7.83 ± 4.02 bc
500 mg·L^−1^ ZnO SMPs	100.00 a	12.08 ± 5.79 a
100 mg·L^−1^ ZnO NPs	100.00 a	6.00 ± 4.29 c
500 mg·L^−1^ ZnO NPs	100.00 a	10.42 ± 3.18 ab
100 mg·L^−1^ ZnO+1%Ag NPs	91.67 a	5.83 ± 4.20 c
500 mg·L^−1^ ZnO+1%Ag NPs	100.00 a	7.58 ± 4.42 bc

Values represent means * or means ± SD **, 12 repetitions included. Means in columns for each cultivar tested followed by the same letter do not differ significantly at *p* ≤ 0.05 (Fisher’s test).

## Data Availability

Data are available by e-mail upon reasonable request.
